# Very High Cycle Fatigue Behavior of Additively Manufactured 316L Stainless Steel

**DOI:** 10.3390/ma13153293

**Published:** 2020-07-24

**Authors:** Boris Voloskov, Stanislav Evlashin, Sarkis Dagesyan, Sergey Abaimov, Iskander Akhatov, Ivan Sergeichev

**Affiliations:** 1 Center for Design, Manufacturing & Materials, Skolkovo Institute of Science and Technology, Bolshoy Boulevard 30, bld. 1, 121205 Moscow, Russia; S.Evlashin@skoltech.ru (S.E.); S.Abaimov@skoltech.ru (S.A.); I.Akhatov@skoltech.ru (I.A.); I.Sergeichev@skoltech.ru (I.S.); 2 Department of Physics, M.V. Lomonosov Moscow State University, Leninskiye Gory 1, bld. 2, 119991 Moscow, Russia; Dagesyan@physics.msu.ru

**Keywords:** additive manufacturing (AM), laser powder bed fusion (L-PBF), very high cycle fatigue (VHCF), fine granular area (FGA), fracture surface, crack initiation

## Abstract

The present paper is focused on an experimental study of the damage-to-failure mechanism of additively manufactured 316L stainless steel specimens subjected to very high cycle fatigue (VHCF) loading. Ultrasonic axial tension-compression tests were carried out on specimens for up to 10^9^ cycles, and fracture surface analysis was performed. A fine granular area (FGA) surrounding internal defects was observed and formed a “fish-eye” fracture type. Nonmetallic inclusions and the lack of fusion within the fracture surfaces that were observed with SEM were assumed to be sources of damage initiation and growth of the FGAs. The characteristic diameter of the FGAs was ≈500 μm on the fracture surface and were induced by nonmetallic inclusions; this characteristic diameter was the same as that for the fracture surface induced by a lack of fusion. Fracture surfaces corresponding to the high cycle fatigue (HCF) regime were discussed as well to emphasize damage features related to the VHCF regime.

## 1. Introduction

Recent studies have improved the quality of additively manufactured (AM) products [1,2,3,4,5]. Despite marked progress, there is a lack of experimental data revealing the mechanisms of damage and failure of structures in real-life complex loading conditions, which represents a critical obstacle for the implementation of additive technologies in the serial production of critical parts [6]. For instance, parts of turbine engines [4,7] and pressure vessels for spallation neutron sources [8,9] that experience from very high cycle fatigue (VHCF) could be additively manufactured.

The properties of the final part highly depend on certain process parameters, such as the laser velocity and power density, scanning strategy, hatch spacing, and thickness of the printed layers [10,11]. The combination of these parameters can be used to fabricate structures with typical defects present in AM metallic parts, such as a lack of fusion, voids, and microcracks induced by residual stresses [12]. Moreover, nonmetallic inclusions can appear within the part, due to imperfections of the feedstock material [13,14,15].

A lack of fusion appears between layers as partially or fully unmelted particles [16] if the scan speed and laser power do not provide the energy density required for normal melting [17]. Moreover, the volumetric heat capacity gradient induced by differences in the size of powder particles results in fusion defects as well [18].

Voids normally arise due to the entrapment of gas bubbles during the laser melting process [17]. However, at a low laser density, voids can be formed due to the entrapment of powder particles if the melt pool is small; therefore, the particles are not molten enough to ensure sufficient bonding [19]. Furthermore, when a combination of the laser power and scan speed exceeds a certain threshold value, the metal is vaporized; such a process generates vapor cavities, collapsing and forming voids [17]. Microcracks caused by the release of a high-stress field induced by a temperature gradient during laser melting also leads to void formation [20]. To diminish those microcracks, hot isostatic pressing (HIP) is applied. Another way to improve the densification of the final part is to increase the overlap of scanning tracks [20] or to preheat the baseplate during the process [21]. All defects mentioned above can cause crack initiation in the VHCF regime [22,23].

Crack initiation and propagation scenarios were proposed for conventional fatigue regimes [24,25,26]. Crack initiation is described as the formation of intrusions and extrusions on the material surface caused by the accumulation of persistent slip bands [27].

However, in the VHCF regime, crack initiation occurs in the core of the material and is accompanied by the growth of so-called fine granular areas (FGAs) in the vicinity of defects [22]. FGAs play a crucial role in the damage that occurs in the VHCF regime [28]; thus, it is assumed that more than 90% of the fatigue life is spent on the formation of FGAs [29]. Sakai has explained that the formation of a FGA occurs in three stages [30]. The first stage is the formation of a fine granular layer as a result of intensive polygonization around the internal defect during very high frequency loading. The second is the nucleation and confluence of microdebonded areas. Lastly, the third stage is the formation of the FGA due to spreading of the microdebonded areas. The model proposed by Murakami et al. [31] explained FGA formation by hydrogen embrittlement caused by trapping hydrogen in the vicinity of internal defects.

The present study investigated the VHCF damage-to-failure mechanism in 316L stainless steel specimens manufactured by laser powder bed fusion (L-PBF). The nonmetallic inclusions and lack of fusion were identified as sources of FGA growth and the formation of a “fish-eye” fracture type. Conventional fatigue tests were performed to emphasize features of material damage in the VHCF regime.

## 2. Materials and Experimental Methods

### 2.1. Materials and Specimens

**Figure 1:**
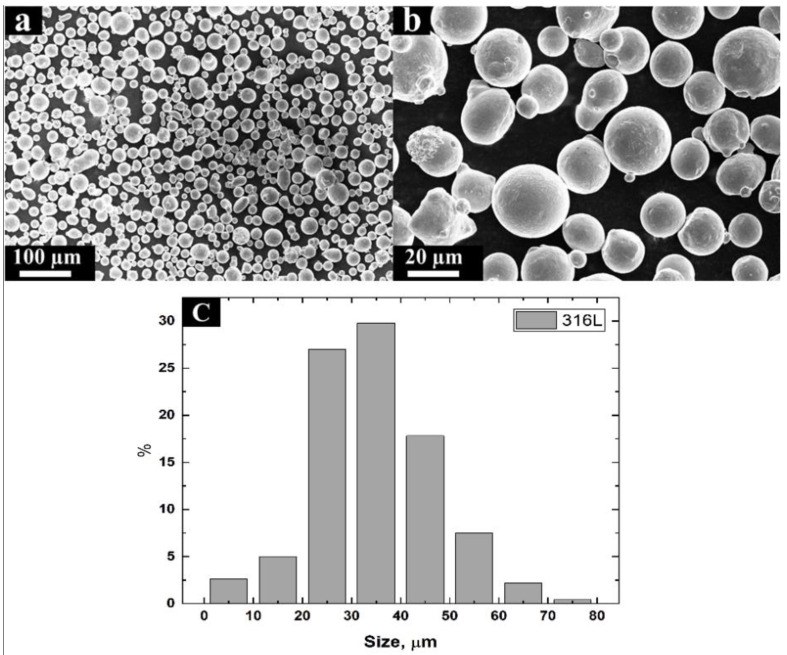
The 316L stainless steel powder used for the L-PBF process, (**a**) magnification ×413; (**b**) magnification ×2000; (**c**) size distribution (the number below the chart shows the medium particle size in the range).

Cylindrical bars, 100 mm in length and 13 mm in diameter, were vertically built by Trumpf TruPrint 1000 (Ditzingen, Germany) facilities, with the process parameters given in Table 1. The laser has a Gaussian profile with a focusing spot of 55 μm. The 316L stainless steel powder particles had a spherical shape with a diameter of 20–50 μm (Figure 1c) [11]. It was observed that some large particles agglomerated with dust-like “satellites” as a result of collisions that were apparently caused by the gas atomization process [32] (Figure 1a,b).

**Figure 2:**
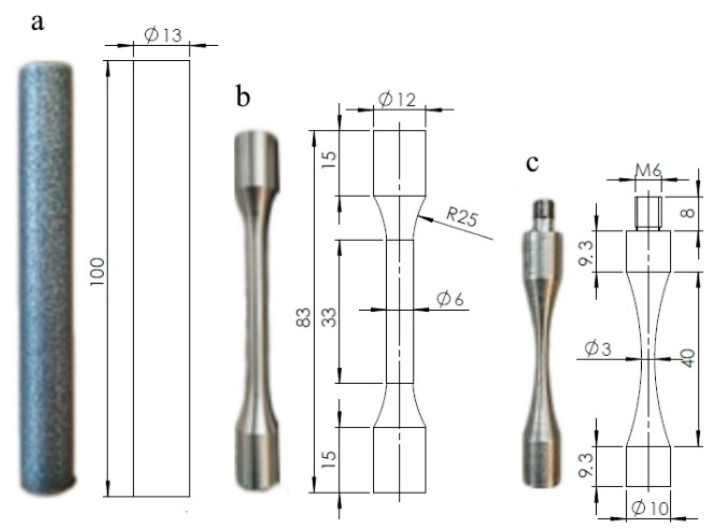
The test specimens with dimensions: (**a**) as-built cylinder; (**b**) a specimen for static and conventional fatigue tests; (**c**) a specimen for VHCF tests. All dimensions shown are in millimeters.

The shapes and dimensions of the printed bars and test specimens milled from the cylinders by the CNC machine are shown in Figure 2. The specimens for the static tensile test were machined to the same shape as conventional fatigue specimens (Figure 2b). No specimens were heat-treated. Static tensile tests were performed at room temperature according to ISO 6892 [33] using an Instron 5969 electromechanical machine with 50 kN load capacity. Five specimens were tested at a loading rate of 2 mm per min. The obtained tensile properties are given in Table 2.

The conventional fatigue tests were performed by an Instron 8801 (Norwood, MA, USA) servo-hydraulic machine with cycle asymmetry coefficient R = 0.1 (R = σ_min_/σ_max_) at a frequency of 30 Hz according to ISO 1099 [34] at room temperature as well. One specimen per stress level was tested.

### 2.2. Ultrasonic Fatigue Tests 

The axial tension-compression VHCF tests were performed using a Shimadzu USF-2000 (Kyoto, Japan) ultrasonic testing machine. Continuous oscillations of the specimen were generated by a piezoelectric actuator with a resonance frequency of 20 kHz ± 30 Hz and a 300 ms pulse. The external frequency supplied by the test machine must be one of the natural frequencies of the specimen. All VHCF specimens were tested at R = −1. FE simulation was performed by SIMULIA Abaqus finite element software (v. 6.14, Dassault Systèmes, Vélizy-Villacoublay, France) to calculate the shape and dimensions of the specimen (Figure 2c), so that its natural frequency is 20 kHz. During the test, the specimens were cooled by dry air to maintain the temperature at the specimen surface between 15–45 °C. The temperature was measured by an infrared thermometer. An eddy current extensometer was used to measure the displacement of the free end of the specimen. The VHCF tests were automatically aborted as soon as the resonance frequency deviated more than 500 Hz from the initial setup frequency due to damage to the specimen. To reveal the fracture surface and keep its morphology after finishing the test, the specimens were statically disrupted at −150 °C in a climate chamber with liquid nitrogen. Then, the fracture surfaces were investigated by scanning electron microscopy (SEM) on a Quattro instrument (Thermo Fisher Scientific, Waltham, MA, USA) equipped with energy-dispersive X-ray spectroscopy (EDX, Bruker XFlash Detector 6, Billerica, MA, USA).

## 3. Results and Discussion

### 3.1. S–N Curves of the Specimens

**Figure 3:**
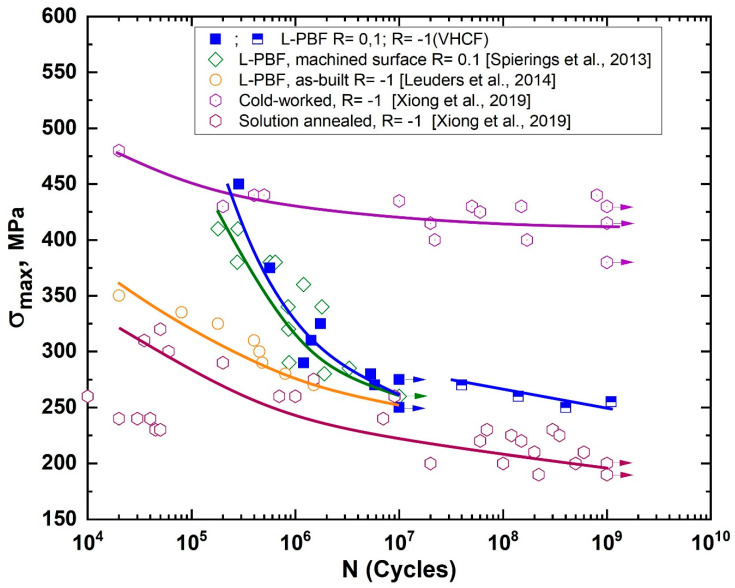
S–N diagram of the L-PBF 316L stainless steel specimens, as well as the data obtained by Spierings et al. [35], Leuders et al. [36], and by Xiong et al. [41] for cold-worked and solution-annealed 316L SS. The points indicated by the arrows correspond to specimens that were not disrupted during the tests.

The fatigue tests were carried out for high and very high cycle fatigue regimes (HCF and VHCF, respectively). The S–N diagrams (maximum cyclic stress versus number of cycles to failure) were obtained, as given in Figure 3. The HCF tests were performed with R = 0.1, and the VHCF tests were performed for fully reversed loading with R = −1. The results of conventional fatigue tests of L-PBF 316L stainless steel obtained by Spierings et al. [35] for R = 0.1 and Leuders et al. [36] for R = −1 are also plotted in Figure 3 and concatenated to the results of the present study. It was seen from the figure that the HCF data obtained herein for R = 0.1 lies within the data given in [35]. However, as recently discussed by Zhang et al. [37], the stress-based transformation of fatigue data from R = 0.1 to R = −1 is questionable for AM 316L stainless steel utilizing traditional models [38,39,40].

As shown in Figure 3, the plotted S–N data corresponding to cold-worked specimens [41] shows higher endurance at similar stress levels than the data in the plot obtained for the AM specimens in HCF [36] and VHCF regimes. However, the AM specimens demonstrated almost the same fatigue behavior as the specimens heat treated at 1120 °C for 7.5 min followed by water quenching [41]. This indicated that the difference could be explained by assuming features of the damage-to-failure mechanism of the AM and routinely manufactured 316L stainless steel under fatigue loading.

### 3.2. Analysis of the Fracture Surfaces

**Figure 4:**
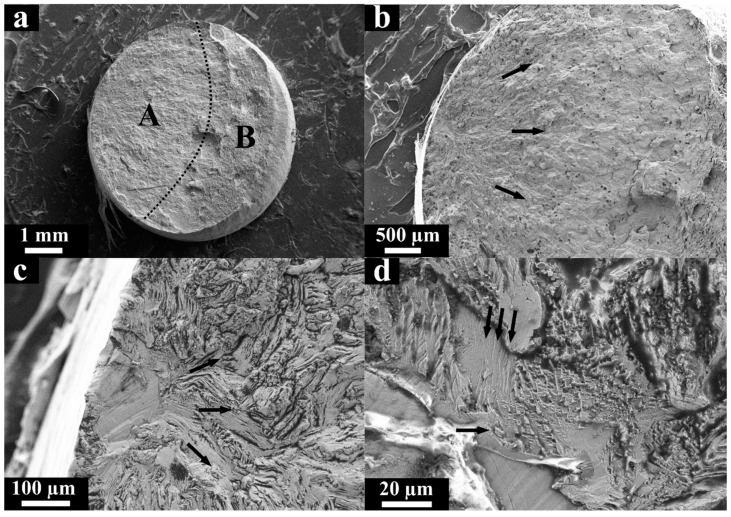
SEM micrographs of the fracture surface of the L-PBF specimen after loading in the high cycle fatigue (HCF) regime (σ_max_ = 310 MPa and N = 1.41 × 10^6^ cycles), (**a**) the characteristic failure zones; (**b**) the direction of the fatigue crack propagation (zone A); (**c**) the crack nucleation site; (**d**) fatigue striations.

Figure 4 shows the fracture surface corresponding to the specimen tested in the HCF regime under average stress level σ_max_ = 310 MPa and N = 1.41 × 10^6^ cycles before rupture. The fracture surface (Figure 4a) consisted of a fatigue crack propagation zone (A) and a final rupture zone (B). The direction of the crack propagation is indicated by arrows in Figure 4b nearest to the outer surface of the specimen where the fatigue crack nucleated (Figure 4c). The fatigue crack nucleation site (Figure 4c) and its propagation path (Figure 4d) are characterized by a rather complicated viscous relief of the fracture surface, caused by a mixed mode that combines shear and detachment. Fatigue striations (Figure 4d) were observed along with the elongated comb formations, which corresponds to the intermittent propagation of the fatigue crack, which is typical for ductile metallic materials. The distance between the individual striations at this stage was approximately 2 μm.

**Figure 5:**
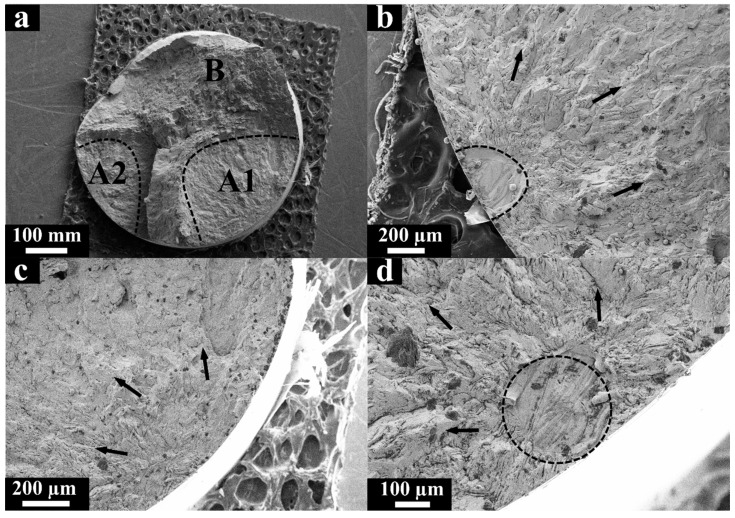
SEM micrographs of the fracture surface of the LPBF specimen after loading in the HCF regime (σ_max_ = 450 MPa and N = 2.23 × 10^5^ cycles), (**a**) zones of failure; (**b**) direction of crack propagation (zone A2); (**c**) the direction of crack propagation (zone A1); (**d**) the site of crack initiation (zone A1).

SEM images of the fracture surface obtained in the HCF regime with the highest stress level σ_max_ = 450 MPa and N = 2.23 × 10^5^ cycles before rupture are given in Figure 5. It was observed that two separate fatigue cracks initiated growth from the outer surface of the specimen (Figure 5b,d). Therefore, two crack propagation zones, A1 and A2, grew until the final rupture at zone B (Figure 5a), which increased according to the applied stress and was proportionally larger than the appropriate rupture zone (Figure 4a) observed in the HCF regime. The sizes of zones A1 and A2 are smaller than the size of zone A produced in the HCF regime (Figure 4a), where the specimen ruptured at low stresses. In other words, the ratio of the crack initiation and propagation zones to the final fracture zone decreased as the stress level increased, which corresponds to previously reported observations [42,43].

Unlike the HCF regime at the average stress level, typical fatigue striations (Figure 4d) were not observed on the fracture surface of the HCF specimen at highest stress level, which is probably related to a higher rate of fatigue crack propagation [42]. Few unmelted particles ≈30 μm in size were detected at the nucleation area where crack propagation zone A2 increased (Figure 5b). At the same time, zone A1 propagated from the outer surface as well, where the AM process defects were not observed. 

**Figure 6:**
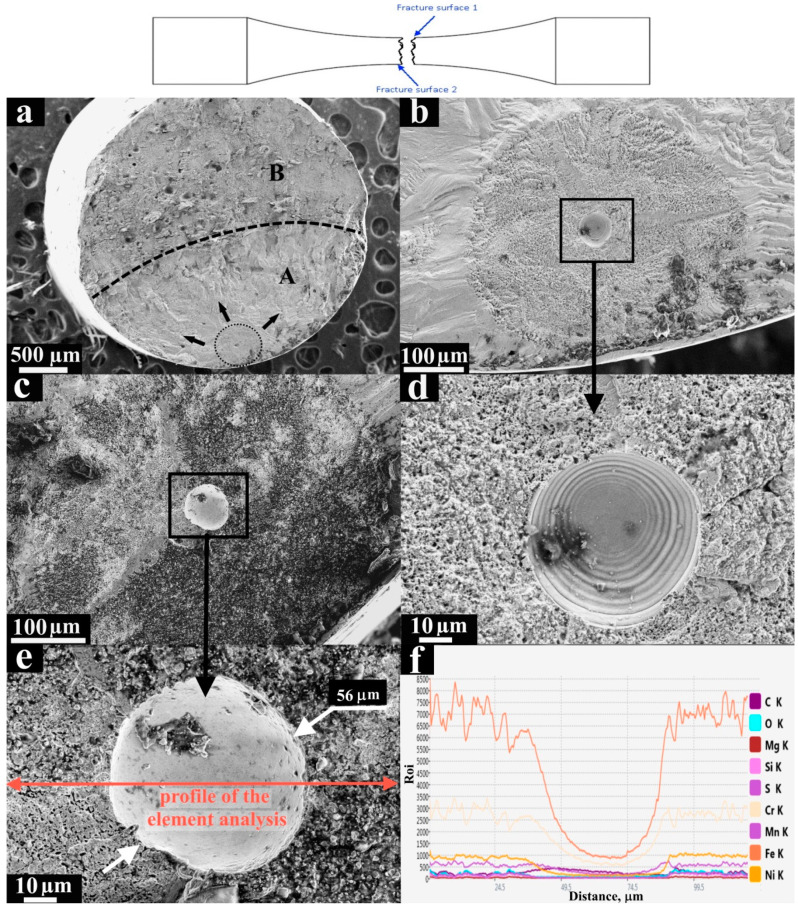
SEM micrographs of the fracture surfaces after loading in the very high cycle fatigue (VHCF) regime (σ_max_ = 270 MPa and N = 4 × 10^7^ cycles) of the L-PBF specimen with the assumed nonmetallic inclusion), (**a**) fracture surface 1; (**b**) FGA and crack initiation site at fracture surface 1; (**c**) crack initiation site at fracture surface 2; (**d**) imprint of the defect at fracture surface 1; (**e**) imprint of the defect at fracture surface 2; and (**f**) element distribution across the defect and its vicinity.

Figure 6 presents the fracture surfaces of the opposite sides of the specimen damaged in the VHCF regime. A fatigue crack (area A at Figure 6a) grew during the ultrasonic test, and then the final brittle rupture (area B at Figure 6a) was achieved by the static tensile test at −150 °C, which minimized the plastic deformation in the cracked cross section to maintain the morphology formed during the VHCF test. The typical VHCF “fish-eye” morphology was obtained surrounding the internal defects (Figure 6b,d), which was confirmed by the imprints of the 56 μm inclusions on the opposite fracture surface of the specimen (Figure 6c,e). The measured size of the FGA was ≈500 μm (Figure 6b). The elemental distribution across the defect and its vicinity (Figure 6e) was determined by EDX mapping analysis. The EDX analysis showed a drop in the concentration of metallic elements and a small growth of carbon and silicon (Figure 6f) in the defect vicinity that enabled the treatment of this defect as a nonmetallic inclusion.

**Figure 7:**
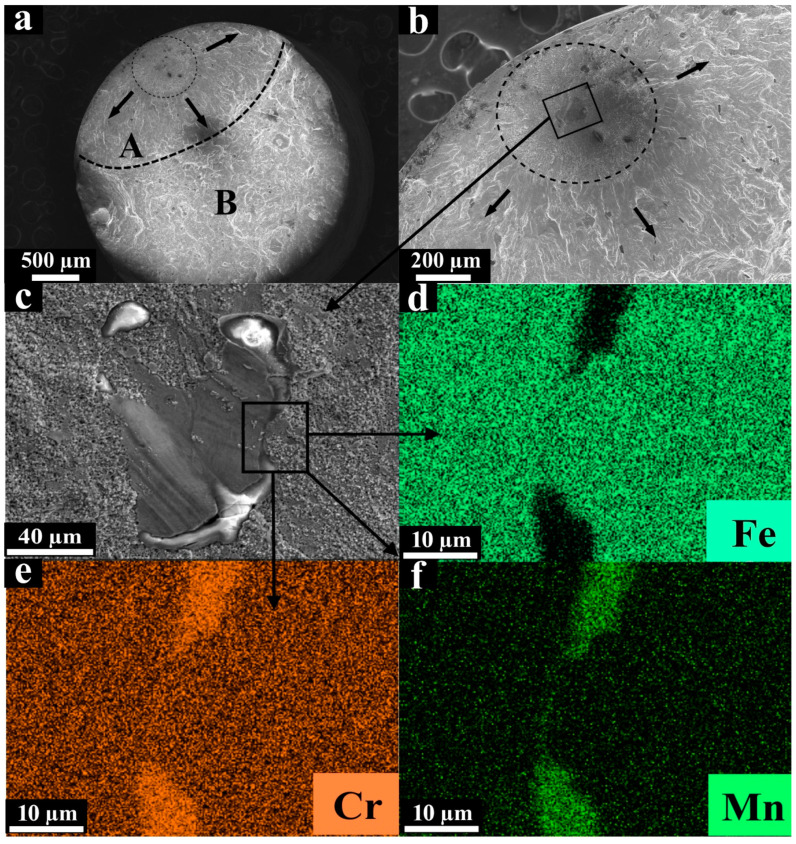
SEM micrographs of the fracture surfaces after loading in the VHCF regime (σ_max_ = 260 MPa, N = 1.5 × 10^8^ cycles) of the L-PBF specimen with the assumed fusion defect, (**a**) zones of failure; (**b**) crack initiation site; (**c**) internal defect; (**d**) iron distribution; (**e**) chromium distribution; (**f**) manganese distribution.

Another characteristic of the VHCF fracture surface is shown in Figure 7a–c. The characteristic size and morphology of the FGA observed there (Figure 7b,c) are similar to those of the FGA surrounding the nonmetallic inclusion mentioned above. Herein, EDX analysis of the defect zone was also performed to identify this type of defect. The boundaries of the defect were enriched by chromium and manganese, and at the same time, a lack of iron was observed there in comparison with the nominal material surrounding the defect. Therefore, the defect was assumed to be an unmelted particle or due to a lack of fusion.

Summarizing the observations above, it is believed that FGAs corresponding to the identified internal defects had a very similar characteristic size and morphology. However, a quantitative analysis of the fracture surfaces shall be performed to describe the FGA morphology, for instance, in terms of scale-invariant properties of the surface roughness [44]. Such analysis will provide a quantitative comparison of the VHCF fracture surface morphology beyond the FGA and the morphology formed in the HCF regimes.

In general, the analysis of fracture surfaces in VHCF regime showed that the crack initiation does not occur on the surface of the material, unlike in the HCF regime. Such behavior is typical for conventionally manufactured metals [22]. However, the imperfections of material caused by the additive manufacturing process can affect the fatigue behavior of the manufactured part.

## 4. Conclusions

The fatigue response and the fracture surface morphology of the additively manufactured 316L stainless steel subjected to VHCF regime were first observed. The obtained herein S–N diagram covering the HCF and VHCF regimes demonstrated the difference between the fatigue behaviors of the AM and conventionally manufactured 316L stainless steel specimens. Particularly, the published S–N data corresponding to cold-worked specimens showed higher endurance at similar stress levels obtained for the AM specimens in the HCF and VHCF regimes. Herein, the AM specimens demonstrated almost the same VHCF behavior as the specimens that were heat treated at 1120 °C for 7.5 min followed by water quenching.

The analysis of the fracture surfaces detected FGAs surrounding internal defects that formed a “fish-eye” fracture type. The nonmetallic inclusions and lack of fusion detected by SEM within the fracture surfaces were assumed to be sources of damage initiation and FGA growth. The ≈500 μm characteristic diameter of the FGA on the fracture surface induced by a nonmetallic inclusion was the same as that for the fracture surface induced by a lack of fusion. It was observed that FGAs corresponding to the identified internal defects had a very similar characteristic size and morphology. However, a quantitative analysis of the fracture surfaces shall be performed to describe the FGA morphology, for instance, in terms of scale-invariant properties of the surface roughness. Such an analysis will provide a quantitative comparison of the VHCF fracture surface morphology beyond the FGA and the morphology formed in the HCF regime.

## Figures and Tables

**Table 1:** The laser powder bed fusion (L-PBF) process parameters.

Laser Power (W)	Power Density (W/cm^2^)	Beam Traverse Speed (mm/sec)	Hatch Spacing (mm)	Layer Thickness (mm)
113	9.51 × 10^6^	600	0.08	0.020

**Table 2:** Tensile properties of the L-PBF 316L specimens.

Elasticity Modulus (GPa)	Yield Strength (MPa)	Ultimate Strength (MPa)	Elongation at Fracture (%)
180 ± 7	479 ± 17	565 ± 12	41 ± 8
